# An incidental situs Inversus Totalis in a Pediatric case of granulomatous Panuveitis. A case report

**DOI:** 10.1093/omcr/omaf228

**Published:** 2025-11-26

**Authors:** Carlos Eduardo Solórzano Flores, Daniel Aguilar, Fabiola Langner-Salinas, Carolina Hovelmeyer Jurgens, Diya Mehta

**Affiliations:** Faculty of Medical Sciences, National Autonomous University of Honduras (UNAH), Calle La Salud, Edificio Administrativo, Tegucigalpa 11101, Honduras; Faculty of Medical Sciences, National Autonomous University of Honduras (UNAH), Calle La Salud, Edificio Administrativo, Tegucigalpa 11101, Honduras; Superior School of Medicine, National Polytechnic Institute of Mexico (IPN), Salvador Díaz Mirón esq. Plan de San Luis S/N, Miguel Hidalgo, Casco de Santo Tomas, Mexico City 07738, Mexico; Faculty of Medicine, Austral University of Chile (UACh), Avenida Independencia 641,Valdivia 5110566, Chile; Alumni, Surat Municipal Institute of Medical Education and Research College, Bombay Market Rd, Sahara Darwaja, Umarwada, Gujarat, 395001, India

**Keywords:** situs inversus totalis, granulomatous panuveitis, congenital, asymptomatic

## Abstract

Situs inversus totalis (SIT) is a rare congenital abnormality characterized by a mirror-image transposition of thoracic and abdominal organs. Although SIT is a recognized congenital anomaly, it is often unfamiliar to physicians because it is typically asymptomatic and discovered incidentally. We report the case of a 7-year-old girl from Central Honduras who presented with decreased visual acuity in the right eye, and was diagnosed with granulomatous panuveitis. Clinical evaluation revealed a right-sided apex beat, systolic murmur, and left-sided hepatic dullness. Electrocardiogram showed right axis deviation, inverted P waves in I and aVL, and positive P waves in aVR. Radiographic and Ultrasonography imaging confirmed dextrocardia, right-sided aortic knuckle, and mirror-image transposition of abdominal organs, consistent with SIT. This rare coexistence of SIT with ocular inflammation highlights the importance of multidisciplinary evaluation in atypical clinical presentations. Although often asymptomatic, SIT requires recognition to avoid diagnostic errors and guide appropriate management.

## Introduction

Situs inversus totalis (SIT) is a rare congenital anatomical condition characterized by a mirror-image reversal of thoracic and abdominal organs, including the heart, lungs, liver, stomach, spleen, and intestines [1] Approximately 0.01% of the general population is affected by this condition [2] and is often discovered incidentally due to its frequently asymptomatic presentation. Although SIT may not always pose direct clinical concerns, it can mislead diagnoses and interventions, particularly in acute settings where standard anatomical orientation is assumed [3].

Most reports focus on the surgical or systemic implications of SIT, while ocular manifestations remain scarcely documented in the literature. This case report contributes to the limited body of knowledge by presenting a pediatric case in which SIT was incidentally diagnosed during the evaluation of granulomatous panuveitis. It highlights the importance of a thorough assessment when managing localized symptoms and underscores the value of recognizing anatomical variants that may influence diagnostic accuracy and treatment strategies.

## Case report

**Figure 1:**
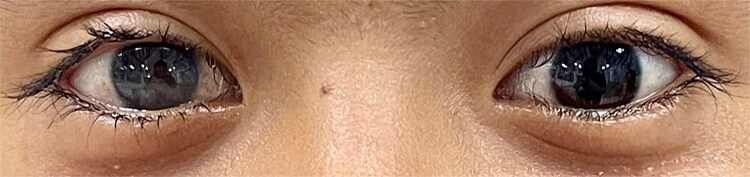
Eyes comparison. Right eye showing a dense central corneal opacity obscuring the iris and pupil, with a relatively clear peripheral cornea and no associated conjunctival injection.

A 7 year old girl from the Lenca community in Comayagua, Honduras, was admitted to the ophthalmology department due to decreased visual acuity in the right eye. (Fig. 1) Slit-lamp examination revealed conjunctival hyperemia with vascular injection and a clear cornea without epithelial staining, but with 360-degree corneal neovascularization. The anterior chamber appeared shallow. Retrokeratic pigment deposits were observed, and the iris was displaced anteriorly, with 360-degree posterior synechiae and areas of iris atrophy. A uveitic cataract was also noted.

Ocular ultrasonography demonstrated organized echoes consistent with membrane formation in the vitreous cavity; the retina was attached with no evidence of detachment on dynamic evaluation. Examination of the left eye was unremarkable. A contrast-enhanced brain MRI was performed to rule out space occupying lesions. There were no significant findings.

Serum IgA, IgG, IgM, and IgE levels, as well as ANA and ANCA, MPO and PR3 were within normal reference ranges. The Mantoux test and serologies for toxoplasmosis, syphilis, and cytomegalovirus (IgM and IgG) were non reactive. However, IgG antibodies against herpes simplex virus type 1 (HSV-1) were markedly elevated (>200 U/mL).

Based on clinical findings, a presumptive diagnosis of chronic granulomatous panuveitis secondary to HSV-1 was made. Slit-lamp and ultrasonography findings supported this impression; however, histopathological confirmation and image documentation were not obtained, reflecting the clinical context and local resource constraints. The diagnosis therefore remained clinical and imaging-based.

The patient was initiated on topical treatment including methylcellulose (1 drop every 6 hours), prednisolone 1% (1 drop daily), a fixed combination of timolol 0.5% and dorzolamide 2% (1 drop daily), and atropine 1% (1 drop daily). She was referred to the pediatric department for further evaluation and systemic workup.

**Figure 2:**
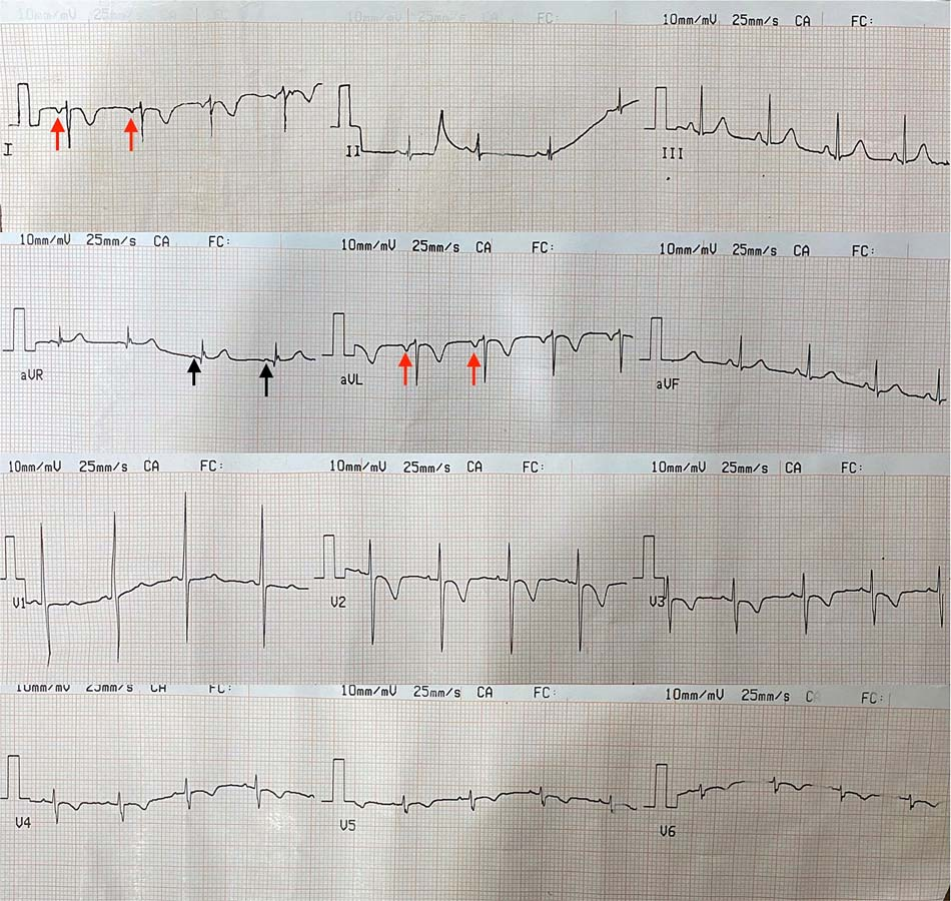
Electrocardiogram. ECG revealing right axis deviation and inverted P waves on leads I, avL (red arrows) and positive on avR (black arrows).

**Figure 3:**
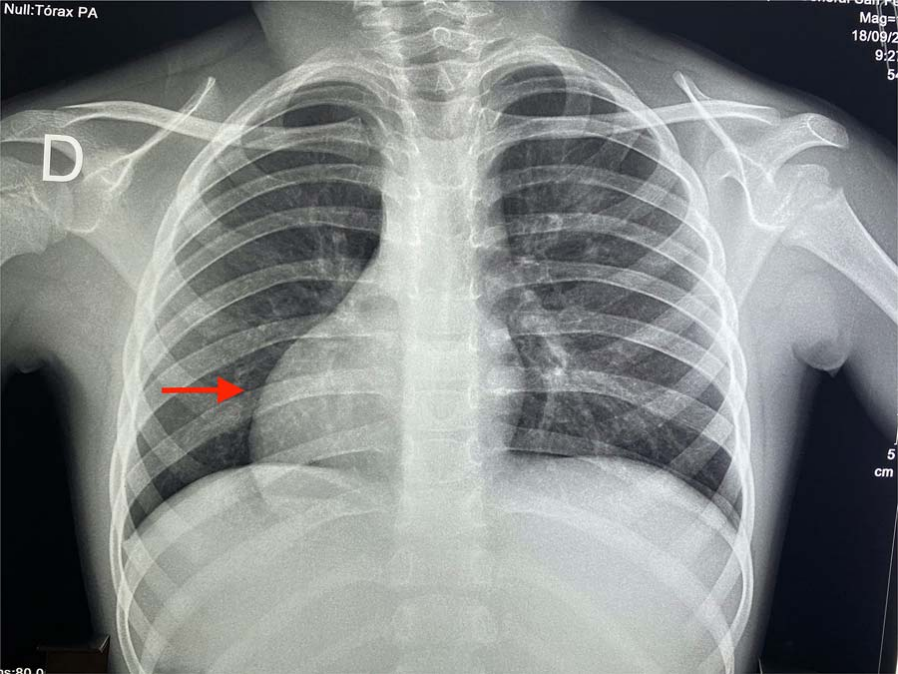
Chest radiograph. Consistent with dextrocardia. (red arrow).

There was no history of recurrent upper or lower respiratory tract infections, nor skin infections. Growth parameters were appropriate for her age, and vital signs were stable. On physical examination, a visible apex beat was noted in the right fifth intercostal space along the midclavicular line, and heart sounds were louder on the right side of the chest. A systolic aortic murmur was heard. Electrocardiogram (ECG) revealed right axis deviation with inverted P waves in leads I and aVL, and positive P waves in aVR (Fig. 2). Abdominal examination revealed no palpable organomegaly; however, percussion indicated hepatic dullness on the left side of the abdomen. A chest radiograph demonstrated findings consistent with dextrocardia, including a right-sided aortic knuckle (Fig. 3).

**Figure 4:**
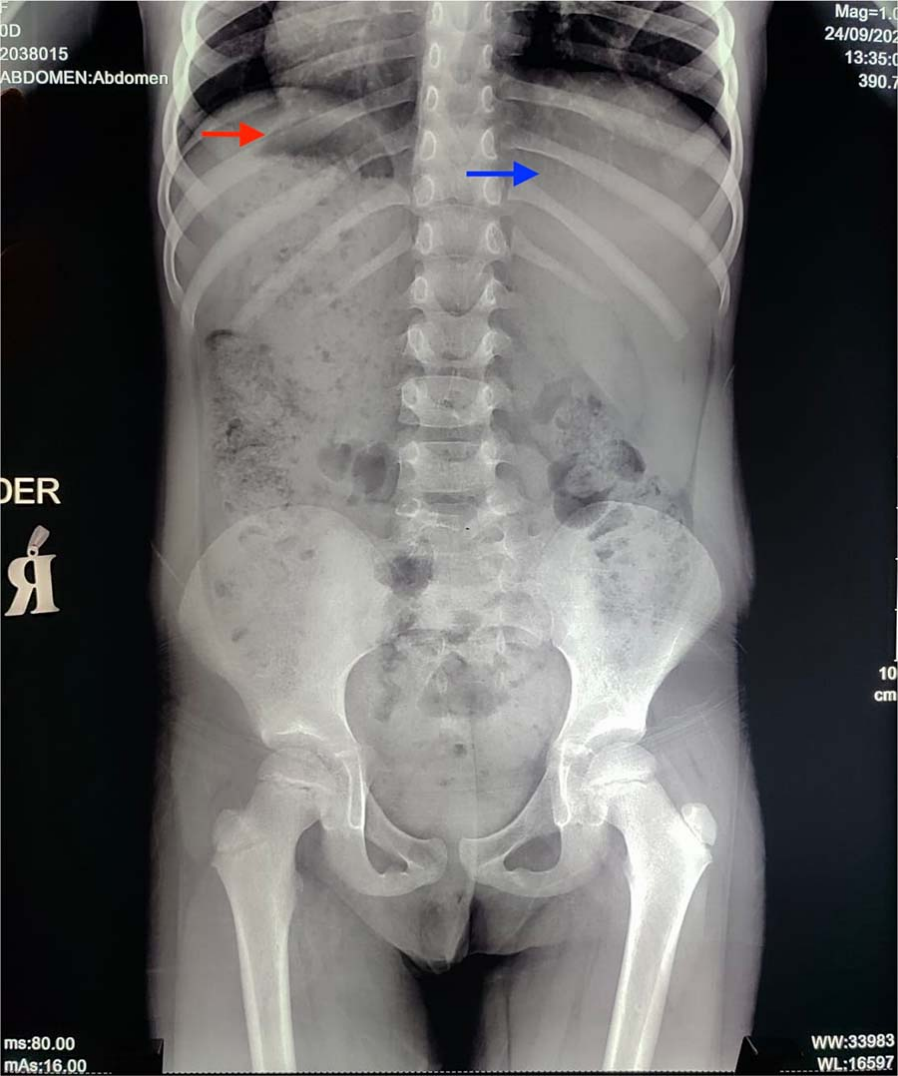
Abdominal radiograph. Consistent with left hepatic shadow (blue arrow) and right gastric bubble (red arrow).

**Figure 5:**
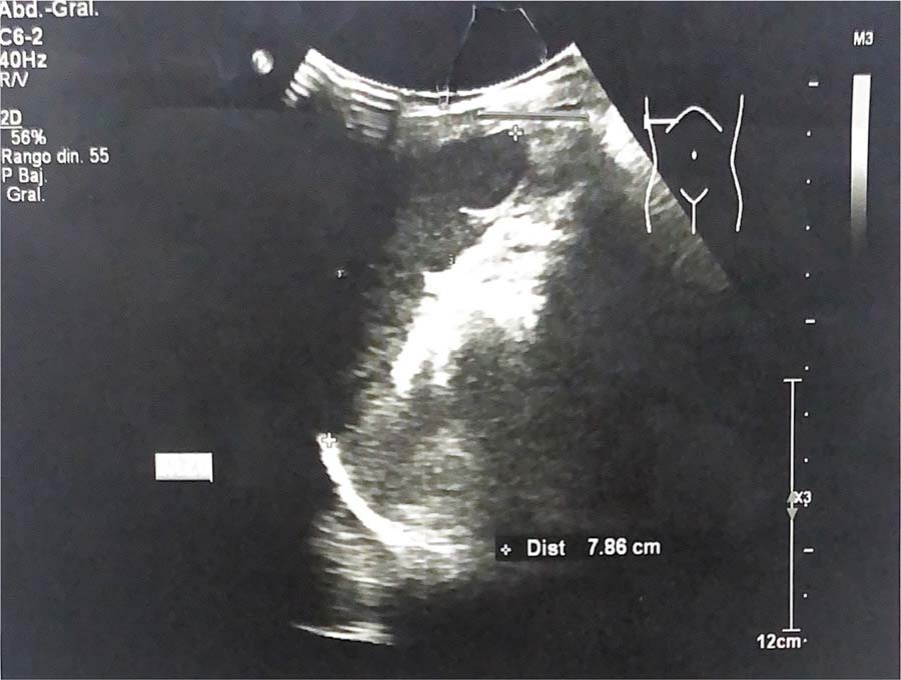
Abdominal ultrasound. Ultrasound image on left upper quadrant showing left sided liver.

An echocardiogram was performed, which showed no structural cardiac defects. Further evaluation included an abdominal radiograph, revealing a left-sided hepatic shadow and a right gastric bubble (Fig. 4). Additionally, abdominal ultrasound demonstrated a mirror-image transposition of the abdominal organs without other abnormalities (Fig. 5). The diagnosis of situs inversus totalis was confirmed. The patient’s parents were counseled and reassured about the findings. On ophthalmologic follow-up examination, the patient reported mild improvement in ocular discomfort, although visual acuity in the right eye remained markedly reduced. No new epithelial defects were observed. The left eye continued to show no abnormalities. Treatment was maintained, and further follow-up was planned to assess inflammatory control and evaluate surgical candidacy.

## Discussion

We report a 7-year-old girl with chronic granulomatous panuveitis in whom situs inversus totalis (SIT) was incidentally identified during systemic evaluation. The coexistence of these findings is exceptionally uncommon, and this case underscores the value of thorough systemic assessment in children presenting with complex ocular inflammation. Ocular features, including unilateral anterior chamber inflammation, keratic precipitates, iris atrophy, posterior synechiae, and a uveitic cataract, together with markedly elevated HSV-1 IgG titers, support a herpesvirus-related etiology, even in the absence of PCR confirmation.

A stepwise approach to differential diagnosis was essential. Infectious causes such as tuberculosis, toxoplasmosis, cytomegalovirus, and syphilis were excluded based on negative serologies, a normal chest radiograph, and a negative Mantoux test. Systemic inflammatory and autoimmune disorders, including juvenile idiopathic arthritis, Behçet disease, Vogt–Koyanagi–Harada syndrome, and inflammatory bowel disease–associated uveitis, were considered but ultimately deemed unlikely given normal immunologic markers (ANA, ANCA, MPO, PR3, IgG, IgM, IgA, IgE) and the absence of systemic manifestations such as arthritis, mucocutaneous lesions, gastrointestinal symptoms, or neurological involvement [4, 5]. Idiopathic granulomatous uveitis remains the most frequent cause of pediatric chronic uveitis, further supporting the plausibility of herpes-related panuveitis in this patient.

Situs inversus totalis is a rare congenital condition affecting roughly 1 in 20 000 births, typically inherited in an autosomal recessive pattern [1]. It arises from disruption of left–right embryonic signaling pathways (including NODAL, LEFTY, and PITX2), resulting in mirror-image organ placement [6, 7]. Most individuals are asymptomatic, although 2–5% may present with additional congenital anomalies, and up to 20% may have primary ciliary dyskinesia (PCD), which can lead to recurrent respiratory infections and reproductive complications [8–10]. SIT affects both sexes, though prevalence is slightly higher in males (1.5:1) [3]. In Kartagener syndrome, males may experience infertility due to defective sperm motility, while females may experience subfertility related to impaired ciliary function in the fallopian tubes. In our patient, no clinical features of PCD were present, and her sex-specific reproductive risks were not clinically relevant; nevertheless, awareness of these differences can inform long term counseling and monitoring [10].

In this patient, echocardiography confirmed dextrocardia without structural cardiac defects, consistent with classic SIT findings [11]. Recognizing such anatomical variants is clinically important, as they may complicate diagnostic interpretation and procedural planning, particularly in acute or surgical settings.

The combination of SIT with pediatric granulomatous panuveitis is rarely described. Most reports of SIT focus on systemic or surgical implications [1–3], and ophthalmologic findings are typically incidental. Some isolated reports suggest possible associations with anatomical variants such as cilioretinal arteries [12]. In contrast, our case highlights a clinically relevant ocular manifestation coexisting with SIT, without evidence of PCD or other syndromic involvement. This observation expands the limited literature and emphasizes that systemic anomalies may be discovered unexpectedly during routine ophthalmologic assessment.

This case reinforces two key points. First, pediatric patients with atypical ocular inflammation benefit from systematic, multidisciplinary evaluation, which can uncover previously unrecognized anatomical anomalies. Second, reporting rare associations such as SIT and granulomatous panuveitis increases awareness among clinicians, aiding accurate diagnosis and appropriate management in similar cases. While histopathological confirmation and clinical imaging were not available, the presumptive diagnosis of granulomatous panuveitis was supported by careful clinical and ultrasonographic evaluation. This report remains relevant, as it highlights the importance of comprehensive systemic assessment in pediatric ocular inflammation, documents the incidental discovery of SIT, and underscores the need for clinician awareness of rare anatomical ocular associations.

Pediatric granulomatous panuveitis may occasionally coincide with rare systemic anomalies such as SIT. Careful clinical assessment, supported by multidisciplinary evaluation, is essential to ensure correct diagnosis and guide management, prevent diagnostic errors, and optimize patient care in complex pediatric ophthalmologic presentations.

## Consent

Written and verbal informed consent was obtained from the patient’s mother, and anonymity and confidentiality were ensured.

## Guarantor

Carlos Solórzano.
